# Clinical Correlates of Premenstrual Suicidal Ideation Among Women in Makkah Population

**DOI:** 10.7759/cureus.68716

**Published:** 2024-09-05

**Authors:** Abdulrahim Gari, Sarah S Almohammdi, Noor s Alharbi, Reema N Alotaibi, Lina AlSulami, Hisham I Isa

**Affiliations:** 1 Department of Obstetrics and Gynecology, Faculty of Medicine, Umm Al-Qura University, Makkah, SAU; 2 Department of Medicine and Surgery, College of Medicine, Umm Al-Qura University, Makkah, SAU

**Keywords:** menstrual health, premenstrual symptoms, prementrual syndrome, suicidal behavior, women’s mental health

## Abstract

Introduction

Suicidal ideation (SI), planning, and attempts have been found to be more common in women with premenstrual syndrome (PMS) and premenstrual dysphoric disorder (PMDD). This study assessed the association between PMS, PMDD, and SI within the Makkah population, in Saudi Arabia.

Methodology

A descriptive cross-sectional study that simply characterizes the prevalence of a health outcome in a specified population was done on 733 women using an online questionnaire. Data about demographics, menstrual cycle, PMS, past psychiatric history, prior history of PMDD, and previous drug treatment for PMDD were collected. The chi-square and Fisher exact tests were applied.

Results

Of the participants, 540 (73.7%) had an age represented the age of 18-25 years old, 551 (75.2%) were single, 592 (80.8%) did not have children, 352 (48.0%) had a four-year college education, and 552 (75.3%) had a monthly income of less than 5000 SR. Exactly 704 (96.0%) were menstruating, 539 (73.5%) had regular menstrual cycles, 640 (87.3%) reported low shock trauma, 201 (27.4%) reported a history of anxiety, and 541 (73.8%) reported an impact of premenstrual disorders on mental health. Of them, 662 (90.3%) experienced premenstrual symptoms, 53 (7.2%) had a previous diagnosis of premenstrual symptoms, and 62 (8.5%) had a history of a suicide attempt. Those aged 18 to 25 years, who have no children, have a monthly income of less than 5000, are unemployed, have irregular menstruation, have medical interventions due to premenstrual symptoms, have a lifelong history of depression or anxiety, and have a history of high shock trauma had a significantly higher prevalence of a history of a suicide attempt. These factors were independent predictors on regression analysis.

Conclusion

The link between PMD and the likelihood of suicide attempts highlights how crucial it is to treat severe PMDs in the context of mental health.

## Introduction

Premenstrual syndrome (PMS) is a recurrent cyclic disorder characterized by moderate to severe physical, emotional, and psychological disturbances that develop particularly during the luteal phase of the menstrual cycle and remit with menstruation [1,2], mainly affecting young and middle-aged women [1]. 

According to The American Congress of Obstetricians and Gynecologists (ACOG), PMS is diagnosed based on the following criteria: the patient must at least experience one affective and somatic symptom five days before the onset of menses in each of the previous three menstrual cycles; affective symptoms include angry outbursts, anxiety, depression, confusion, irritability, social withdrawal, somatic symptoms include headache, abdominal bloating, breast tenderness or swelling, swelling of extremity, weight gain, and joint or muscle swelling [3,4]. Although the definitive ethology has not yet been identified, several studies suggest that it is due to increased sensitivity to normal hormonal changes of estrogen and progesterone, along with neurotransmitter abnormality like serotonin, which triggers the symptoms [5].

An estimated 90% of women who were fertile experienced premenstrual symptoms, ranging from moderate to severe. Of them, between 20% and 40% have PMS, and between 2% and 8% have premenstrual dysphoric disorder (PMDD) [6]. The disease burden can be high, as women with PMS experience dysfunction in social or economic performances, with higher rates of absenteeism, higher medical utilization, and affected quality of life [3]. Women with PMS and PMDD have been linked to increased suicidal ideation (SI), plans, and attempts [7,8]. 

A recent study examined the co-occurrence of premenstrual symptoms and SI in 300 individuals. A total of 27% of those who experienced premenstrual symptoms reported experiencing new or worsened SI. The study found significant differences between women with premenstrual symptoms and SI (PMSI) and women without PMSI; women with PMSI were more likely to be unmarried, unemployed, and not parenting. They also reported that premenstrual symptoms significantly interfered with their lives and had a higher prevalence of psychiatric hospitalizations, traumatic childhood experiences, suicidal thoughts, and depression and anxiety 9 [9]. 

In a case-control study of 120 fertile, regularly menstruating women admitted to a hospital after attempting suicide, compared to a control group of 120 women from different medical wards, PMDD was identified through a mental health interview based on DSM-5 criteria, the study found. Suicidal attempts and PMDD may be related, but the menstrual cycle is unrelated to PMDD. Suicidal behaviors and PMS have no connection to one another [10]. Additionally, according to recent meta-analysis and systematic reviews, women experiencing moderate-to-severe premenstrual disorders may be considered high risk groups for suicide and should be screened for suicidality [8,11]. 

Although PMS and PMDD are prevalent gynecological conditions that can lead to significant complications and negatively impact an individual's quality of life, no research has yet been conducted in Saudi Arabia addressing the correlation between PMS, PMDD, and SI. Therefore, this study investigates the connection between PMS, PMDD, and SI within the Makkah population.

## Materials and methods

Study design and setting

In order to determine the prevalence of illnesses in clinic-based samples and for population-based surveys, a descriptive cross-sectional study was conducted. They are usually less expensive and can be completed somewhat more quickly. The current study was done in Makkah City, Saudi Arabia. 

Study participants 

The study targeted all women aged 18 to 55 years in Makkah City, Saudi Arabia, who agreed to participate in this study, and all met the inclusion criteria. Male gender, females aged 55 years, women who are pregnant, and participants who disagree with participating in this study were excluded.

Sample size

The Raosoft online sample size calculator was used to calculate the sample size. Based on a margin of error of 5%, a confidence interval (CI) of 95%, and a Saudi female population of 15.39 million, the minimum calculated sample was 385 participants.

Study instrument

A validated self-administered questionnaire from the previous study was distributed electronically via social media applications after it was 12 translated into Arabic and directed to women living in Makkah City, Saudi Arabia, during the study period [9]. After being created in English,, the questionnaire was translated into Arabic. A back-translation procedure was used to guarantee the translation's validity and accuracy. After then, any differences between this back-translation and the original English text were found and fixed. The accuracy and intent of the original questionnaire were faithfully maintained in the Arabic translation, thanks to this approach. Cronbach's alpha (0.78) for the questionnaire indicated strong reliability, and the results held true after multiple administrations. Three experts assessed the questionnaire validity, where the item received a score of "4" if it was adequate (simple, relevant, and clear); "3" if it was adequate but needed minor adjustment; "2" if it required considerable change; and "1" if it wasn't so adequate (might be omitted). The content validity index (CVI) is the proportion of all items that expert’s rate as a 3 or 4. A score of 80% on the CVI is considered to have strong validity. After establishment, the proposed questionnaire's CVI was determined to be 91.2%.

The questionnaire contains three sections. The first section asked about sociodemographic data like age, marital status, educational level, and income. The second section contained two parts. The first part collected information about the menstrual cycle and PMS, such as menstrual regularity, menstrual status, and screening of PMS symptoms. The second part asked about past psychiatric history, such as previous psychiatric diagnosis, current mood, anxiety symptoms, and depression symptoms. Finally, the third section of this questionnaire included prior history of PMDD and previous drug treatment for PMDD. 

Ethical part and confidentiality

Before the participants started answering the questionnaire, their informed consent was sought. Participants were allowed to leave the survey at any time throughout the research process. Participants were not asked for any information that might expose their identity, and confidentiality and privacy were maintained. The ethical approval for this study was from Umm Al-Qura University Institutional Research Board with reference no. HAPO-02-K-012-2023-10-1827. 

Data analysis 

The main goal of this research is to investigate the connection between PMS, PMDD, and SI within the Makkah population. Both descriptive and inferential statistical analysis of the data was carried out. Simple frequencies and percentages of the categorical variables were calculated and tabulated. To find the association between various factors including PMS and PMDD with suicidal attempts, chi square test and Fisher exact test were applied. Fischer exact test was used where the expected count in cells was less than 5. Statistical significance was established at a p-value of 0.05 or less with a 95% CI. Multivariate logistic regression analyses was done to assess factors associated with SI. The odds ratio (OR) was calculated at a 95% CI. All the statistical calculations were performed using the IBM SPSS Statistics for Windows, Version 27 (Released 2020; IBM Corp., Armonk, New York, United States).

## Results

In this study, a total of 733 participants completed the study questionnaire. The most represented age group was 18-25 years old, comprising 540 (73.7%) of the participants. The majority of respondents were single, with 551 (75.2%) reporting single status. As for parenthood, 592 (80.8%) did not have children. Exactly 352 (48.0%) had four-year college (Bachelor of Arts, Bachelor of Science) and 266 (36.3%) had secondary certificates. A monthly income of less than 5000 SR was reported among 552 (75.3%) participants, while 63 (8.6%) had monthly income exceeding 10000 SR. More details about the sociodemographic information are provided in 15 (Table 1).

**Table 1:** Sociodemographic characteristics of the participants in the study (n = 733)

		Frequency	%
Age	18 to 25	540	73.7%
	26 to 35	94	12.8%
	36 to 45	68	9.3%
	46 to 55	31	4.2%
Marital status	Single	551	75.2%
	Married	166	22.6%
	Divorced	11	1.5%
	Widowed	5	0.7%
Children?	Yes	141	19.2%
	No	592	80.8%
Educational qualification	Four-year college (Bachelor of Arts, Bachelor of Science)	352	48.0%
	Secondary certificate	266	36.3%
	Masters	22	3.0%
	PHD degree	7	1.0%
	General education certificate	36	4.9%
	Less than a high school diploma	19	2.6%
	Two-year college (equivalent degree)	24	3.3%
	Professional degree (Doctorate in Medicine, Doctorate in Law)	7	1.0%
Income rate:	Less than 5000	552	75.3%
	5000-10,000	118	16.1%
	More than 10,000	63	8.6%
Occupation:	Housewife	98	14.0%
	Full-time work	90	12.9%
	Part-time work	28	4.0%
	Not employed	482	69.1%

Table 2 presents the clinical characteristics of the participants involved in the study. Exactly 704 (96.0%) reported menstruating, 539 (73.5%) had regular menstrual cycles, 640 (87.3%) reported low shock trauma, and 201 (27.4%) of the participants reported a history of anxiety. The impact of premenstrual disorders on various aspects of the participants' lives was notable. Mental health was affected for a significant majority (541; 73.8%).

**Table 2:** Clinical characteristics of the participants in the study (n = 733)

		Frequency	%
Menstrual condition	Menstruate	704	96.0%
	She doesn't menstruate	29	4.0%
Menstrual regularity	Regular	539	73.5%
	Irregular	194	26.5%
Having a history of trauma	High shock	93	12.7%
	Low shock	640	87.3%
Participants with a history of			
Depression		111	15.1%
Anxiety		201	27.4%
Psychiatric hospitalization		51	7.0%
Medical interventions due to premenstrual symptoms		42	5.7%
Drug treatment for premenstrual dysphoric disorder		58	7.9%
Hormonal contraception		139	19.0%
Premenstrual disorder impact on participants			
Physical health and physical strength		420	57.3%
Daily activities		525	71.6%
Mental health		541	73.8%
Social life		326	44.5%
Surroundings		285	38.9%
Participants whose daily activities are affected by psychological symptoms		495	67.5%

In Table 3, exactly 662 (90.3%) reported experiencing premenstrual symptoms. A total of 53 (7.2%) had previous diagnosis of premenstrual symptoms. Furthermore, 62 (8.5%) had a history of a suicide attempt. Also, 56 (7.6%) of the participants had premenstrual symptoms and suicide attempt, while 10 (1.3%) had a previous diagnosis of premenstrual disorder and suicide attempt.

**Table 3:** Prevalence of PMS, PMDD, and suicide attempts among participants PMS: Premenstrual syndrome; PMDD: premenstrual dysphoric disorder

	Frequency	%
Premenstrual symptoms:	662	90.3%
Previous diagnosis of premenstrual disorder	53	7.2%
Having a history of a suicide attempt	62	8.5%
Women with premenstrual symptoms and suicide attempt	56	7.6%
Women with previous diagnosis of premenstrual disorder and Suicide attempt	10	1.3%

In Table 4, among participants with a history of suicide attempts, exactly 56 (90.3%) reported the presence of PMS. Strikingly, 606 (90.3%) of the participants without suicide attempts also experienced PMS. Additionally, among those diagnosed with PMDD, exactly 10 (16.1%) reported a history of suicide attempts, and 43 (6.4%) did not have a history of suicide attempts with a recorded statistical significance of p = .010.

**Table 4:** Association of PMS, PMDD, and suicide attempts PMS: Premenstrual syndrome; PMDD: premenstrual dysphoric disorder

		Having a history of a suicide attempt				Sig.
		Yes		No		
		Frequency	Column N %	Frequency	Column N %	
Presence of premenstrual symptoms	Yes	56	90.3%	606	90.3%	0.998
	No	6	9.7%	65	9.7%	
Having a previous diagnosis of premenstrual disorder	Yes	10	16.1%	43	6.4%	0.010*
	No	52	83.9%	628	93.6%	
*p < 0.05, significant						

Table 5 shows that 54 (10.0%) participants aged 18-25 years had a significant association with history of suicide attempts versus one (1.5%) of others aged 36-45 or more with recorded statistical significance (p = .021). Also, 56 (9.5%) of the participants without children reported a higher percentage of suicide attempts versus not having children and a history of suicide attempts with recorded statistical significance (p = .046). A total of 56 (10.1%) participants with income less than 5000 had a significant association with the history of suicide attempts with recorded statistical significance (p = .014). A total of 49 (10,2%) unemployed participants had a significant association (p = .049) with a history of suicide attempts. Moreover, 30 (15.5%) of the participants with irregular menstrual cycle had a significant association (p = .001) with history of suicide attempts versus 32 (5.9%) participants with regular menstrual cycle. Additionally, participants who underwent medical interventions due to premenstrual symptoms had a lifelong history of depression, anxiety, or experienced high shock trauma and displayed significantly higher percentages of suicide attempts among the affected individuals (all p = .001).

**Table 5:** Association of various sociodemographic and clinical factors with suicide attempts

		Having a history of a suicide attempt				Sig.
		Yes		No		
		Frequency	Row N %	Frequency	Row N %	
Age	18 to 25	54	10.0%	486	90.0%	0.021*
	26 to 35	7	7.4%	87	92.6%	
	36 to 45	1	1.5%	67	98.5%	
	46 to 55	0	0.0%	31	100.0%	
Marital status	Single	53	9.6%	498	90.4%	0.305
	Married	9	5.4%	157	94.6%	
	Divorced	0	0.0%	11	100.0%	
	Widowed	0	0.0%	5	100.0%	
Children?	Yes	6	4.3%	135	95.7%	0.046*
	No	56	9.5%	536	90.5%	
Educational qualification	Four-year college (Bachelor of Arts, Bachelor of Science)	34	9.7%	318	90.3%	0.910
	Secondary certificate	22	8.3%	244	91.7%	
	Masters	0	0.0%	22	100.0%	
	PHD degree	0	0.0%	7	100.0%	
	General education certificate	3	8.3%	33	91.7%	
	Less than a high school diploma	1	5.3%	18	94.7%	
	Two-year college (equivalent degree)	2	8.3%	22	91.7%	
	Professional degree (Doctorate in Medicine, Doctorate in Law)	0	0.0%	7	100.0%	
Income rate	Less than 5000	56	10.1%	496	89.9%	0.014*
	5000-10,000	3	2.5%	115	97.5%	
	More than 10,000	3	4.8%	60	95.2%	
Occupation	Housewife	3	3.1%	95	96.9%	0.049*
	Full time work	4	4.4%	86	95.6%	
	Part time work	2	7.1%	26	92.9%	
	Not employed	49	10.2%	433	89.8%	
Menstrual condition	Menstruate	60	8.5%	644	91.5%	1.000
	She doesn't menstruate	2	6.9%	27	93.1%	
Menstrual regularity	Regular	32	5.9%	507	94.1%	<0.001*
	Irregular	30	15.5%	164	84.5%	
The presence of medical interventions due to premenstrual symptoms	Yes	12	28.6%	30	71.4%	<0.001*
	No	50	7.2%	641	92.8%	
Having a lifelong history of depression	Yes	38	34.2%	73	65.8%	<0.001*
	No	24	3.9%	598	96.1%	
Having a lifelong history of anxiety	Yes	43	21.4%	158	78.6%	<0.001*
	No	19	3.6%	513	96.4%	
Having a history of trauma	High shock	27	29.0%	66	71.0%	<0.001*
	Low shock	35	5.5%	605	94.5%	
*p < 0.05, significant						

Multivariate logistic regression analysis was done to assess the risk factors (independent predictors) of having a history of suicide attempt among studied participants. It was found that having a previous medical intervention due to premenstrual symptoms, having a lifelong history of depression or anxiety, or having a history of high shock trauma was a risk factor (independent predictors) of having a history of suicide attempt among the studied participants (Table 6).

**Table 6:** Multivariate logistic regression analysis of risk factors of having a history of a suicide attempt

Variable	B	Wald	p-value	Odds ratio (CI: 95%)
Age	0.65	2.19	0.138	1.92 (0.8-4.58)
Children?	0.75	0.92	0.335	2.13 (0.45-9.99)
Income rate	0.51	1.85	0.173	1.67 (0.79-3.52)
Occupation	0.24	1.27	0.258	0.77 (0.5-1.2)
Menstrual regularity	0.49	2.18	0.139	0.61 (0.31-1.17)
The presence of medical interventions due to premenstrual symptoms	1.25	7.01	0.008	3.5 (1.38-8.87)
Having a lifelong history of depression	1.66	20.02	<0.001	5.26 (2.54-10.91)
Having a lifelong history of anxiety	1.03	7.04	0.008	2.81 (1.31-6.04)
Having a history of trauma	1.32	13.82	<0.001	3.76 (1.87-7.58)

## Discussion

Our study aimed to investigate the connection between PMS, PMDD, and SI within the Saudi population.

Menstrual cycle characteristics

The high prevalence of regular menstruation (73.5%) among studied participants resonates with Park et al., who observed a similar distribution in their study on menstrual regularity [12]. The substantial prevalence of premenstrual symptoms among participants (90.3%) underscores the pervasive nature of this phenomenon. These findings align with Gao et al.'s investigation, which reported a similar high prevalence of premenstrual symptoms in their study population [6]. The widespread occurrence of symptoms emphasizes the need for increased awareness and targeted interventions to alleviate the burden experienced by a significant majority. A relatively smaller percentage (7.2%) of participants had received a formal diagnosis of premenstrual disorder, suggesting a potential underdiagnosis or lack of recognition within healthcare settings. This echoes the findings of Chan et al., who highlighted the challenges in accurately diagnosing premenstrual disorders [13]. 

Psychiatric history

The prevalence of high shock trauma (12.7%) among studied women echoes the work of Kulkarni et al., supporting the notion that a majority of individuals typically exhibit resilience in the face of trauma [14]. The prevalence of mental health conditions, particularly depression (15.1%) and anxiety (27.4%), emphasizes the intersection between mental health and menstrual disorders. These figures align with Padhy et al., suggesting a consistent association between mental health conditions and premenstrual symptoms [15]. The reported history of psychiatric hospitalizations (7.0%) and medical interventions for premenstrual symptoms (5.7%) indicates the clinical severity of premenstrual disorders. These findings are congruent with Luggin et al.'s work, highlighting the need for targeted interventions in severe cases [16]. The utilization of hormonal contraception by 19.0% of the participants reflects a noteworthy aspect of managing premenstrual symptoms. This finding resonates with Bäckström et al.'s study, which emphasizes the role of hormonal interventions in mitigating premenstrual distress [17].

The substantial impact on various life domains, including physical health, daily activities, mental health, social life, and surroundings, highlights the multidimensional burden of premenstrual disorders. These outcomes align with recent research by Liguori et al., underscoring the pervasive impact of premenstrual symptoms on diverse aspects of individuals’ lives [18]. The significant impact of psychological symptoms on daily activities (67.5%) echoes the work of Anderson et al., who emphasized the impairment caused by psychological aspects of premenstrual disorders [19]. This aligns with the broader understanding of how psychological symptoms can profoundly affect functional aspects of daily life. 

Prevalence of a history of suicide attempts

The reported history of suicide attempts among participants in the current study was 8.5%. This result agrees with that observed in a recent Saudi study done in 2024 which found that SI, plan, and attempt had respective lifetime prevalence rates of 4.90 %, 1.78 %, and 1.46 % and 12-month prevalence rates of 1.82 %, 0.89 %, and 0.63 %. The study found that significant correlates of STB include younger age, female gender, singe marital status, and prior mental disorders [20]. The low diagnosis rate of premenstrual disorder among studied females (7.2%) indicates a critical gap in identifying and addressing this condition, calling for improved screening and diagnostic practices. The premenstrual SI raises concerns about the mental health implications of premenstrual disorders. This finding resonates with the work of Prasad et al.'s systemic review, who identified a correlation between severe premenstrual symptoms and an increased risk of SI [8]. This review included 13 studies and revealed that women with PMDD are almost seven times at higher risk of suicide attempt and almost four times as likely to exhibit SI. Similarly, women with PMS are also at increased risk of SI [8].The acknowledgment of suicide attempts underscores the gravity of mental health implications associated with premenstrual disorders. The co-occurrence of premenstrual symptoms and suicide attempts in 7.6% of the participants highlights a concerning intersection of menstrual health and mental health. Previous literature also suggested that women with PMS and PMDD exhibit higher rates of suicidality when compared with women without these diagnoses [10, 21,22]. This finding also aligns with recent research by Dózsa-Juhász et al., which emphasized the need for comprehensive mental health assessments in individuals presenting with severe premenstrual symptoms [23]. The identification of such comorbidities is crucial for developing targeted interventions that address both the menstrual and mental health aspects of individuals affected. In the case of participants with a formal diagnosis of premenstrual disorder and a history of suicide attempts (1.3%), this subgroup warrants particular attention in clinical practice. The studies addressing this specific intersection necessitate further investigation to understand the unique challenges and risk factors associated with this population. The observation that 90.3% of participants, both with and without a history of suicide attempts, reported premenstrual symptoms suggests that the mere presence of these symptoms alone may not be directly indicative of an increased risk of suicide attempts. This finding aligns with the work of Raval et al., who similarly found a high prevalence of premenstrual symptoms in both clinical and nonclinical populations, emphasizing the complexity of this relationship [24]. 

The specific association between diagnosed premenstrual disorder and a heightened likelihood of suicide attempts aligns with the growing body of literature emphasizing the severe impact of clinically significant premenstrual conditions on mental health. This finding resonates with the recent work of Prasad et al., who underscored the importance of early identification and intervention in individuals diagnosed with premenstrual disorders, particularly those at risk for adverse mental health outcomes [7]. The observed significant association between age and a history of suicide attempts aligns with existing research, such as the work of Osborn et al., which identified a higher prevalence of suicidal behaviors among younger individuals [10]. 

The decreasing prevalence with age suggests a potential age-related vulnerability, highlighting the need for targeted interventions for the younger age group. The notable association between not having children and a higher prevalence of suicide attempts among participants echoes the findings of Knifton et al., who reported increased mental health challenges among individuals without children. This connection may reflect the influence of social support and family dynamics on mental health outcomes [25].

The findings of this study have several significant clinical implications. The high prevalence of premenstrual symptoms and their impact on various aspects of the participants’ lives underscores the importance of increased awareness and early intervention. Healthcare providers should be vigilant in recognizing and addressing the multifaceted nature of premenstrual disorders, considering both clinical and sociodemographic factors. Additionally, the association between diagnosed premenstrual disorder and a heightened risk of suicide attempts emphasizes the need for mental health assessments and targeted interventions for individuals diagnosed with severe premenstrual conditions. Furthermore, the sociodemographic factors associated with suicide attempts, such as age, parental status, income, and occupation, call for tailored interventions that address specific vulnerabilities within these subgroups.

Limitations

The study has several limitations that should be acknowledged. The reliance on self-reported data introduces the possibility of recall bias, as participants may not accurately recall or report their experiences. Additionally, the cross-sectional nature of the study design limits our ability to establish causation and discern the temporal relationships between variables. Furthermore, a selection bias may be present due to the online recruitment method. The study’s sample composition, while diverse, may not fully represent the broader population, and the findings may not be generalizable to individuals from different cultural or socioeconomic backgrounds. Furthermore, the study did not explore potential confounding factors that could influence the associations observed, such as the use of specific medications or the presence of other medical conditions. The study’s focus on certain clinical and sociodemographic factors may omit relevant variables that could contribute to a more comprehensive understanding of premenstrual disorders. 

## Conclusions

This study found that 7.2% of studied females had a previous diagnosis of premenstrual symptoms and 8.5% had a history of a suicide attempt. Sociodemographic factors, including age, parental status, income, and occupation, were identified as potential risk factors for suicide attempts. The association between diagnosed premenstrual disorder and a heightened risk of suicide attempts emphasizes the clinical significance of recognizing and addressing severe premenstrual conditions within a mental health context and highlights the importance of considering diverse vulnerabilities within specific subgroups.

## Appendices

Study questionnaire

You are kindly invited to take part in a research study conducted by medical students from Umm AL-Qura University and supervised by Dr Abdulrahim Gari.

The aim of this study is to investigate the connection between PMS, PMDD, and SI within the Makkah population. Your involvement in the research will be highly valuable in aiding us in further investigation. The study guarantees complete anonymity; thus, there is no need for you to disclose your name or any personal identifying details.

The principles of voluntary participation, nonharm to participants, and maintaining anonymity and confidentiality are firmly upheld in this study. We sincerely appreciate your participation in this study. The online survey is expected to take approximately three minutes to complete.

Questionnaire

**Table 7:** Questionnaire to assess prevalence and severity of PMS and PMDD, their potential links to suicidal thoughts, and associated factors such as social, economic, and psychiatric aspects PMS: Premenstrual syndrome; PMDD: premenstrual dysphoric disorder

Question	Response
Consent	Yes/no
The first part of the questionnaire gathered data on the sociodemographic characteristics of the participants	
Age	18 to 25/26 to 35/36 to 45/46 to 55/+55
Marital status	Single/married/committed relationship/widowed/divorced
Has biological children?	Yes/no
Education	high school degree/college/bs/professional/jd
Income	<5000/5000-10000/>10000
Employment	Homemaker/unemployed/employed occasionally/employed part-time/employed full time
The second part collected information about menstrual status and premenstrual symptoms	
Menstrual status	Regular periods/irregular periods
Premenstrual symptoms	Yes/no
Interference from premenstrual symptoms	Yes/no
Additionally, the participants were asked about past psychiatric diagnosis, current mood and anxiety symptoms, past stressors	
Lifetime history of depression	Yes/no
Lifetime history of anxiety	Yes/no
History of suicide attempt	Yes/no
History of psychiatric hospitalization	Yes/no
Trauma history	High trauma /low trauma
Also, the questionnaire evaluated the premenstrual symptoms screening: Have you ever had a year when most of your periods included at least one of the following symptoms in the week leading up to your period?	
Feeling depressed, sad, down, blue, hopeless, worthless, or guilty?	Yes/no
Feeling anxious, tense, or on edge?	Yes/no
Sudden mood swings or more sensitivity to what others say or do?	Yes/no
Feeling angry or irritable?	Yes/no
Feeling less interested in your usual activities?	Yes/no
Difficulty concentrating?	Yes/no
Feeling lethargic, tired, fatigued, or lacking in energy?	Yes/no
Having an increased appetite, overeating, or food cravings?	Yes/no
Sleeping more than usual, napping, difficulty getting out of bed, or difficulty falling or staying asleep?	Yes/no
Feeling overwhelmed, unable to cope, or out of control?	Yes/no
Physical symptoms such as breast tenderness, breast swelling, a bloated sensation, weight gain, headache, or joint or muscle pain?	Yes/no
Finally, the participants were asked if they had previously been diagnosed with PMDD and previous drug treatment for PMDD?	
Previous hormonal contraceptives?	Yes/no
Previous diagnosis of PMDD?	Yes/no
Previous drug treatment for PMDD?	Yes/no
Previous psychological treatment for PMDD?	Yes/no
Do psychological symptoms affect your daily activities?	Yes/no

## References

[1] Akbulut Ö, Jafari L, Aygün Arı D, Pehlivantürk Kızılkan M, Derman O, Akgül S (2024). Prevalence of premenstrual syndrome in adolescent girls. Turk J Pediatr.

[2] Gudipally PR, Sharma GK (2024). Premenstrual Syndrome. https://www.ncbi.nlm.nih.gov/books/NBK560698/.

[3] Kuehner C, Nayman S (2021). Premenstrual exacerbations of mood disorders: findings and knowledge gaps. Curr Psychiatry Rep.

[4] Ducasse D, Jaussent I, Olié E, Guillaume S, Lopez-Castroman J, Courtet P (2016). Personality traits of suicidality are associated with premenstrual syndrome and premenstrual dysphoric disorder in a suicidal women sample. PLoS One.

[5] Hantsoo L, Epperson CN (2015). Premenstrual dysphoric disorder: epidemiology and treatment. Curr Psychiatry Rep.

[6] Gao M, Zhang H, Gao Z, Cheng X, Sun Y, Qiao M, Gao D (2022). Global and regional prevalence and burden for premenstrual syndrome and premenstrual dysphoric disorder: a study protocol for systematic review and meta-analysis. Medicine (Baltimore).

[7] Chan JH, Lo C, Hsu CD (2021). Premenstrual dysphoric symptoms and lifetime suicide experiences in patients with mood disorder. Gen Hosp Psychiatry.

[8] Prasad D, Wollenhaupt-Aguiar B, Kidd KN, de Azevedo Cardoso T, Frey BN (2021). Suicidal risk in women with premenstrual syndrome and premenstrual dysphoric disorder: a systematic review and meta-analysis. J Womens Health (Larchmt).

[9] Carlini SV, Weiss SJ, Mordukhaev L, Jacob S, Flynn HA, Deligiannidis KM (2022). Clinical correlates of women endorsing premenstrual suicidal ideation: a cross-sectional study. Biopsychosoc Med.

[10] Shams-Alizadeh N, Maroufi A, Rashidi M, Roshani D, Farhadifar F, Khazaie H (2018). Premenstrual dysphoric disorder and suicide attempts as a correlation among women in reproductive age. Asian J Psychiatr.

[11] Osborn E, Brooks J, O'Brien PM, Wittkowski A (2021). Suicidality in women with premenstrual dysphoric disorder: a systematic literature review. Arch Womens Ment Health.

[12] Park YJ, Shin H, Jeon S, Cho I, Kim YJ (2021). Menstrual cycle patterns and the prevalence of premenstrual syndrome and polycystic ovary syndrome in Korean young adult women. Healthcare (Basel).

[13] Chan K, Rubtsova AA, Clark CJ (2023). Exploring diagnosis and treatment of premenstrual dysphoric disorder in the U.S. healthcare system: a qualitative investigation. BMC Womens Health.

[14] Kulkarni J, Leyden O, Gavrilidis E, Thew C, Thomas EH (2022). The prevalence of early life trauma in premenstrual dysphoric disorder (PMDD). Psychiatry Res.

[15] Padhy SK, Sarkar S, Beherre PB, Rathi R, Panigrahi M, Patil PS (2015). Relationship of premenstrual syndrome and premenstrual dysphoric disorder with major depression: relevance to clinical practice. Indian J Psychol Med.

[16] Luggin R, Bernsted L, Petersson B, Jacobsen AT (1984). Acute psychiatric admission related to the menstrual cycle. Acta Psychiatr Scand.

[17] Bäckström T, Andreen L, Birzniece V (2003). The role of hormones and hormonal treatments in premenstrual syndrome. CNS Drugs.

[18] Liguori F, Saraiello E, Calella P (2023). Premenstrual syndrome and premenstrual dysphoric disorder's impact on quality of life, and the role of physical activity. Medicina (Kaunas).

[19] Indusekhar R, Usman SB, O'Brien S (2007). Psychological aspects of premenstrual syndrome. Best Pract Res Clin Obstet Gynaecol.

[20] Altwaijri Y, Benjet C, Al-Habeeb A (2024). Suicidal thoughts and behaviors in the Kingdom of Saudi Arabia. J Affect Disord.

[21] Pilver CE, Libby DJ, Hoff RA (2013). Premenstrual dysphoric disorder as a correlate of suicidal ideation, plans, and attempts among a nationally representative sample. Soc Psychiatry Psychiatr Epidemiol.

[22] Wittchen H -U, Becker E, Lieb R, Krause P (2002). Prevalence, incidence and stability of premenstrual dysphoric disorder in the community. Psychol Med.

[23] Dózsa-Juhász O, Makai A, Prémusz V, Ács P, Hock M (2023). Investigation of premenstrual syndrome in connection with physical activity, perceived stress level, and mental status-a cross-sectional study. Front Public Health.

[24] Raval CM, Panchal BN, Tiwari DS, Vala AU, Bhatt RB (2016). Prevalence of premenstrual syndrome and premenstrual dysphoric disorder among college students of Bhavnagar, Gujarat. Indian J Psychiatry.

[25] Knifton L, Inglis G (2020). Poverty and mental health: policy, practice and research implications. BJPsych Bull.

